# Estrogens Protect Calsequestrin-1 Knockout Mice from Lethal Hyperthermic Episodes by Reducing Oxidative Stress in Muscle

**DOI:** 10.1155/2017/6936897

**Published:** 2017-09-10

**Authors:** Antonio Michelucci, Simona Boncompagni, Marta Canato, Carlo Reggiani, Feliciano Protasi

**Affiliations:** ^1^CeSI-MeT, Center for Research on Ageing and Translational Medicine, University G. d'Annunzio of Chieti, 66100 Chieti, Italy; ^2^Department of Neuroscience, Imaging, and Clinical Sciences (DNISC), University G. d'Annunzio of Chieti, 66100 Chieti, Italy; ^3^Department of Biomedical Sciences, University of Padova, 35131 Padova, Italy; ^4^Department of Medicine and Aging Science (DMSI), University G. d'Annunzio of Chieti, 66100 Chieti, Italy

## Abstract

Oxidative stress has been proposed to play a key role in malignant hyperthermia (MH), a syndrome caused by excessive Ca^2+^ release in skeletal muscle. Incidence of mortality in male calsequestrin-1 knockout (CASQ1-null) mice during exposure to halothane and heat (a syndrome closely resembling human MH) is far greater than that in females. To investigate the possible role of sex hormones in this still unexplained gender difference, we treated male and female CASQ1-null mice for 1 month, respectively, with Premarin (conjugated estrogens) and leuprolide (GnRH analog) and discovered that during exposure to halothane and heat Premarin reduced the mortality rate in males (79–27% and 86–20%), while leuprolide increased the incidence of mortality in females (18–73% and 24–82%). We then evaluated the (a) responsiveness of isolated muscles to temperature and caffeine, (b) sarcoplasmic reticulum (SR) Ca^2+^ release in single fibers, and (c) oxidative stress and the expression levels of main enzymes involved in the regulation of the redox balance in muscle. Premarin treatment reduced the temperature and caffeine sensitivity of EDL muscles, normalized SR Ca^2+^ release, and reduced oxidative stress in males, suggesting that female sex hormones may protect mice from lethal hyperthermic episodes by reducing both the SR Ca^2+^ leak and oxidative stress.

## 1. Introduction

Calsequestrin-1 (CASQ1) is an acidic protein which binds Ca^2+^ with moderate affinity, but high capacity, localized in the lumen of sarcoplasmic reticulum (SR) in proximity of ryanodine receptor type-1 (RYR1), the Ca^2+^ release channel of the SR [1–5]. CASQ1 and RYR1 are two main players in excitation-contraction (EC) coupling, the mechanism that links the depolarization of the transverse tubule (TT) membrane to the release of Ca^2+^ from the SR terminal cisternae which, in turn, activates muscle contraction [6]. In EC coupling, CASQ1 plays the dual role of intraluminal Ca^2+^ buffer and modulator of RYR1-mediated SR Ca^2+^ release [7–10].

We previously demonstrated that CASQ1 knockout mice (CASQ1-null) are vital under normal conditions [11], although the ablation of CASQ1 causes structural and morphological rearrangement of SR membranes at the triad junction, SR depletion, and abnormalities in SR Ca^2+^ release [11–14]. Interestingly, we also discovered that CASQ1-null mice trigger lethal hyperthermic episodes when exposed to both halothane and heat [15, 16], a phenotype that closely resembles that observed in *porcine stress syndrome* (*PSS*) [17, 18], and in knockin mice carrying point mutations in RYR1 gene linked to human malignant hyperthermia (MH) susceptibility, the RYR1^Y522S/WT^ and RYR1^R163C/WT^ mice [19, 20]. MH is a potentially lethal disorder triggered in humans by administration of halogenated/volatile anesthetics (i.e., halothane and isofluorane) and characterized by hyperthermia, rhabdomyolysis (i.e., the rupture of muscle fibers), and increased plasma/serum levels of K^+^, Ca^2+^, and creatine kinase (CK) [21, 22]. The widely accepted molecular mechanism underlying these crises is that the triggering agents, commonly used during surgery interventions, trigger a sustained and uncontrolled release of Ca^2+^ from the SR of skeletal muscle fibers [23, 24].

In Dainese et al. [15], we also showed that incidence of mortality in CASQ1-null mice during exposure to both halothane and heat is significantly greater in males than in females, a finding in line with some epidemiological studies conducted in humans, which reported a male prevalence of ~3 : 1 to 4 : 1 [25–27]. To date, the reason for this gender difference in humans and in CASQ1-null mice remains unclear.

Durham et al. showed that in RYR1^Y522S/WT^ mice the enhanced production of oxidative species of oxygen and nitrogen (ROS and RNS, resp.) results in RYR1 S-nitrosylation/glutathionylation, covalent modifications of RYR1 which further increase the opening probability of the mutated channel [28]. These findings suggested that excessive Ca^2+^-dependent production of ROS/RNS likely plays a pivotal role in the cellular and molecular events leading to rhabdomyolysis of muscle fibers during MH reactions. In line with these findings, in Michelucci et al., [29] we reported that treatment of CASQ1-null male mice with antioxidants (i.e., N-acetylcysteine or Trolox) markedly reduced the rate of heat- and halothane-induced mortalities.

In the current study, we hypothesized that the difference in MH susceptibility between male and female CASQ1-null mice could reside in their different abilities to modulate oxidative stress. Indeed, it is well documented that female sex steroid hormones, that is, estrogens, have potent cellular antioxidant properties [30, 31], thanks to (i) their capability to upregulate the expression of several antioxidant enzymes; (ii) downregulate ROS-generating enzymes [32–34]; and (iii) their direct free-radical scavenging properties [35]. To investigate the possible role that estrogens play in gender difference in CASQ1-null mice, we treated for 1 month (3 to 4 months of age) male CASQ1-null mice with Premarin (a mixture of water-soluble-conjugated equine estrogens) and female CASQ1-null mice with leuprolide (a synthetic nonapeptide that functions as a potent gonadotropin-releasing hormone, or GnRH, analogue), to abolish estrogens production. Results of our experiments indicate that Premarin and leuprolide treatments reverse the halothane- and heat-induced mortalities of CASQ1-null mice, where Premarin exerted a striking protective effect in males while leuprolide increased significantly the MH susceptibility of females. Investigation of the possible molecular mechanisms indicates that estrogens reduce both SR Ca^2+^ leak and oxidative stress, two key events in MH crises.

## 2. Materials and Methods

### 2.1. Ethic Statement

All *in vivo* experiments/protocols on animals were conducted according to the Directive of the European Union 2010/63/UE and the National Institutes of Health Guide for the Care and Use of Laboratory Animals and approved by the Committee on the Ethics of Animal Experiments of the University of Chieti (15/2011/CEISA/COM).

### 2.2. Premarin and Leuprolide Treatments of CASQ1-Null Mice

CASQ1-null mice were generated as previously described [11]. Mice were housed in microisolator cages at 20°C in a 12 hrs light/dark cycle and provided free access to water and food. Three-month-old male and female CASQ1-null mice were randomly assigned to one of the three different experimental groups: control group, Premarin-treated male mice, and leuprolide-treated female mice.

Premarin (Wyeth Laboratories, Dallas, TX, USA) was dissolved in 0.9% NaCl solution and administered subcutaneously to CASQ1-null male mice at a final dose of 40 ng/g of body weight every day for 1 month. Leuprolide acetate salt (Sigma-Aldrich, Italy) was dissolved in 0.9% NaCl solution and also administered subcutaneously to CASQ1-null female mice at a final dose of 100 ng/g of body weight every day for 1 month.

### 2.3. Halothane Exposure and Heat Stress Protocol

To determine MH susceptibility to volatile halogenated anesthetics, mice were exposed to an air mixture containing halothane (Sigma-Aldrich, Italy) at concentrations sufficient to induce stage 3 anesthesia (2% halothane, with more added as necessary to induce and maintain this level of anesthesia) using an Isotec-3 evaporator (GE Healthcare, Milan, Italy), as previously described [15, 29]. During halothane exposure, mice were kept in a chamber at a constant temperature (32°C) to avoid a drop in body temperature during anesthesia. The maximum exposure time to halothane was 1 hr, and surviving mice were then recovered by suspension of anesthetic administration.

To determine MH susceptibility to high environmental temperature, mice were subjected to a heat stress protocol, performed in an environmental chamber at 41°C for 1 hr while their internal temperature was recorded, as previously described [15, 29]. Core body temperature was measured using a rectal thermometer (four channels thermometer TM-946, XS instruments, Modena, Italy) taped on the tails of the animals and recorded every 5 min throughout the duration of heat challenge, as in [15, 29].

### 2.4. Assessment of Rhabdomyolysis

#### 2.4.1. Histologic Analysis

Immediately after heat stress, *extensor digitorum longus* (EDL) muscles were carefully dissected from CASQ1-null mice and fixed at room temperature in 3.5% glutaraldehyde 0.1 M Na cacodylate buffer, pH 7.2 overnight. Small bundles of fixed fibers were then postfixed in 2% OsO_4_ in the same buffer for 2 hrs and then block-stained in aqueous saturated uranyl acetate. After dehydration, specimens were embedded in an epoxy resin (Epon 812). Semithin (800 nm) histological sections were cut with a Leica Ultracut R Microtome (Leica Microsystem, Vienna, Austria) using a Diatome diamond knife (Diatome Ltd., Biel, Switzerland). After staining with toluidine blue dye, the sections were viewed using a Leica DMLB light microscope (Leica Microsystem, Vienna, Austria). The percentage of fibers presenting signs of rhabdomyolysis was determined as previously described [29].

#### 2.4.2. Immunofluorescence Analysis

EDL muscles were carefully dissected immediately after the heat stress protocol and fixed with 2% paraformaldehyde for 2 hrs, at room temperature. Small bundles of fixed fibers were processed for confocal microscopy (CM) acquisitions as previously described [36]. Briefly, samples were first exposed to a mouse monoclonal primary antibody which recognizes both RYR1 and RYR3 (34C, 1 : 20; Developmental Studies Hybridoma Bank, University of Iowa) and then to a Cy3 goat anti-mouse IgG secondary antibody (Jackson ImmunoResearch Laboratories, West Grove, PA, USA). Images were acquired using a LSM510 META laser scanning confocal microscope system (Zeiss, Jena, Germany) equipped with Zeiss Axiovert 200 inverted microscope and a Plan Neofluar oil-immersion objective (63X/1.3 NA).

#### 2.4.3. Quantitative Plasma and Serum Analyses

For quantitative assessment of CK, K^+^, and Ca^2+^ blood/serum markers of rhabdomyolysis, blood samples were collected from anesthetized mice following a brief exposure (30–35 min) to a nontriggering heat stress challenge. Blood samples were collected and processed as previously described [29]. Briefly, mice were anesthetized and 500–800 *μ*l of blood was collected from the right ventricle with a 26-G needle. Approximately, half of this volume was placed in vials containing lithium heparin to prevent blood clotting and centrifuged at 2500*g* (4°C for 15 min) to isolate plasma. The other half of the blood was placed in a vial without anticoagulant, and serum was obtained by centrifugation at 4000*g* (4°C for 20 min). Spectrophotometrical measurements were performed using a Screen Touch Master spectrophotometer (Hospitex Diagnostic, Sesto Fiorentino, Italy).

### 2.5. *Ex Vivo* and *In Vitro* Experiments in Isolated Muscles and Single Fibers

#### 2.5.1. Temperature and Caffeine Sensitivity of Intact EDL Muscles

Intact EDL muscles were dissected from hind limbs of mice, placed in a dish containing Krebs-Henseleit (KH) solution, pinned, and tied with fine silk sutures at each end. Muscles were then mounted vertically between two platinum electrodes immersed in an organ chamber filled with KH solution and attached to a servomotor and force transducer (model 1200A, Aurora Scientific, Aurora, ON, Canada). Before starting the experimental protocol, stimulation level and optimal muscle length (*L_0_*) were determined using a series of 80 Hz tetani in order to adjust the muscle to the length that generated maximal force (*F_0_*). Twitch and tetanic contractile properties, as well as force-frequency parameters, were measured and analyzed. During the experiments, temperature was kept constant at 25°C. To evaluate the development of contractures induced by increasing temperature, EDL muscles were electrically stimulated with a series of consecutive twitches (1 ms duration, 0.2 Hz for each twitch) applied every 5 seconds and exposed to an increase in temperature of 2°C every 5 min (from 25°C to 41°C) [15]. To determine caffeine sensitivity of resting tension and caffeine-dependent decay in twitch force, muscles were subjected to an *in vitro* contracture test (IVCT) protocol as previously described [29]. Briefly, while isolated EDL muscles were continuously stimulated at 0.2 Hz at 23–25°C, caffeine concentration in the bath was changed every 3 min (no wash between applications) as follows: 2, 4, 6, 8, 10, 14, 18, and 22 mM.

#### 2.5.2. Cytosolic Ca^2+^ Measurements in Isolated Single FDB Fibers

Myoplasmic Ca^2+^ transients (60 Hz, 2 s) and caffeine-induced Ca^2+^ release were measured in fibers isolated from *flexor digitorum brevis* (FDB) according to a modified collagenase/protease method described previously [37]. Twenty-four hours after dissociation, FDB fibers were incubated with 5 *μ*M Fura-2 acetoxymethyl ester (Fura-2 AM; Invitrogen, Eugene, OR, USA) for 30 min at 37°C, in a buffer containing the following: 125 mM NaCl, 5 mM KCl, 1 mM MgSO_4_, 1 mM KH_2_PO_4_, 5.5 mM glucose, 1 mM CaCl_2_, 20 mM HEPES, and 1% bovine serum albumin, pH 7.4. A minimum of 30 min was allowed for Fura-2 deesterification before the fibers were imaged. Intracellular Ca^2+^ transients were recorded at 25°C using a dual-beam excitation fluorescence photometry setup (IonOptix Corp., Milton, MA, USA), as previously described [11, 12]. Single fibers were subjected to 2 different stimulation protocols. (a) To evaluate tetanic transients, two trains of high-frequency stimulation (60 Hz for 2 s) were delivered with a recovery time of 5 min between trains. Fura-2 ratios were calculated and analyzed using IonWizard software (IonOptix Corp., Milton, MA, USA). Peak amplitude was calculated by subtracting basal fluorescence ratio values from peak ratio values. (b) To evaluate myoplasmic Ca^2+^ transients at increasing caffeine concentrations, fibers were continuously stimulated with a series of low frequency (0.5 Hz) trains in the presence of 10 mM of caffeine.

### 2.6. Measurements of Oxidative Stress Levels

#### 2.6.1. Glutathione Assay

Reduced and oxidized levels of glutathione (GSH and GSSG, resp.) were measured as previously described [29]. Briefly, hind limb muscles were homogenized, and total GSH and GSSG levels were measured according to Rahman et al. [38]. 0.1 g of tissue from the hind limb muscles was homogenized and centrifuged, and intracellular GSH and GSSG levels were measured as previously described [39]; the assay was performed in 96-well plates (96 Well Tissue Culture Test Plate; Spl Life Sciences, Korea) using an Absorbance Microplate Reader SpectraMAX 190 (Molecular Devices, Sunnyvale, CA, USA). Data were normalized to a GSH standard curve with the GSH concentration in the samples determined from a linear regression from the GSH standard curve [38]. All reagents for these experiments were purchased from Sigma-Aldrich (Milan, Italy).

### 2.6.2. Western Blot Analyses

EDL muscles were homogenized in a lysing buffer containing 3% sodium dodecyl sulphate (SDS) (Sigma-Aldrich, Milan, Italy) and 1 mM EGTA (Sigma-Aldrich, Milan, Italy), using a mechanical homogenizer, and then centrifuged for 15 min at 900*g* at room temperature. Protein concentration was determined spectrophotometrically using a modified Lowry method. 20–40 *μ*g of total protein was resolved in 10–12% polyacrylamide electrophoresis gels, transferred to nitrocellulose membrane, and blocked with 5% nonfat dry milk (EuroClone, Milan, Italy) in Tris buffered saline and Tween 20 0.1% (TBS-T) for 1 hr. Membranes were then probed with primary antibodies diluted in 5% nonfat dry milk in TBS-T overnight at 4°C: (a) anticopper/zinc superoxide dismutase (SOD1) antibody (rabbit polyclonal 1 : 2000, Santa Cruz Biotechnology, Inc., Dallas, TX, USA); (b) antimanganese superoxide dismutase (SOD2) antibody (rabbit polyclonal, 1 : 5000; Santa Cruz Biotechnology, Inc., Dallas, TX, USA); (c) anti-3-nitrotyrosine (3-NT) antibody (mouse monoclonal, 1 : 500; Merck Millipore, Italy); (d) antineuronal nitric oxide synthase (nNOS) antibody (mouse monoclonal, 1 : 2000; BD Biosciences, Milan, Italy); (e) antiNADPH oxidase gp91phox membrane-bound subunit (NOX2) antibody (rabbit monoclonal, 1 : 3000; Abcam, Cambridge, UK); and (f) anticatalase (CAT) antibody (mouse monoclonal, 1 : 1000; Santa Cruz Biotechnology, Inc., Dallas, TX, USA). The antiglyceraldehyde-3-phosphate dehydrogenase (GAPDH) antibody (mouse monoclonal, 1 : 5000; OriGene Technologies, Inc., Rockville, MD, USA) was used as a loading control. Membranes were then incubated with the secondary antibody horseradish peroxidase-conjugated (1 : 10000, Merck Millipore, MA, USA), diluted in 5% nonfat dry milk in TBS-T, for 1 hr at room temperature. Proteins were detected by enhanced chemiluminescent liquid (Perkin-Elmer, Milan, Italy). Protein quantification was made using ImageJ software (National Institutes of Health, Bethesda, MA, USA).

### 2.7. Statistical Analyses

Statistical significance in experiments of halothane- and heat-induced mortalities was evaluated using a two-tailed Fisher's exact test. One-way ANOVA followed by post hoc Tukey test was used for statistical analyses of all other experiments except for the *in vivo* core temperature and the ex vivo temperature and caffeine sensitivity, in which statistical significance was determined using a repeated measures ANOVA followed by post hoc Tukey test for the pairwise comparisons. In all cases, differences were considered statistically significant at *p* < 0.05. Two-tailed Fisher's exact test was performed using GraphPad software, whereas one-way ANOVA and repeated measures ANOVA were performed using Origin 8.0 software.

## 3. Results

### 3.1. Estrogens Protect CASQ1-Null Mice from Halothane- and Heat-Induced Sudden Death by Reducing Hyperthermia

At four months of age, male and female control mice and mice treated with Premarin (males) or leuprolide (females) were exposed to either halothane (2%, 1 h at 32°C) or heat stress protocol (41°C, 1 h), as previously done in [15, 29].

**Figure 1:**
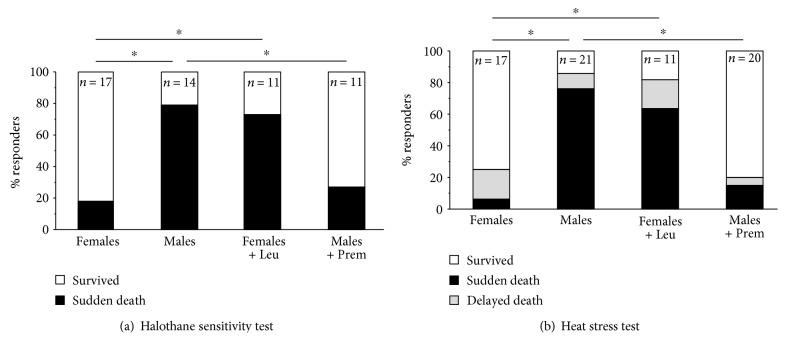
Mortality rate during exposure to halothane and heat stress protocols. Incidence of sudden and delayed (i.e., within 24 hrs after challenge) deaths during exposure to halothane (2% for 1 hr (a)) and to heat (41°C for 1 hr (b)) in male and female CASQ1-null mice, either untreated or treated with Premarin (males) or leuprolide (females). ^∗^*p* < 0.05; *n* = number of mice. See also Tables S1 and S2.

Consistent with the previous results [15], in CASQ1-null mice, the mortality rate, during the administration of halothane and during heat stress protocol, was significantly lower in female mice (18% and 24%) than in male mice (79% and 86%) (Figure 1 and Supplementary Tables 1 and 2 available online at https://doi.org/10.1155/2017/6936897), with a male prevalence of ~4 : 1. The halothane and heat-induced hyperthermic crises exhibited a clinical presentation very similar to that observed during a classic anesthetic-induced MH reaction in humans: difficulty in breathing, tachypnea, impaired movements, and diffuse skeletal muscle rigidity (visual observation). Treatment of mice resulted in a clear reversion of the phenotype (Figure 1): Premarin had a striking protective effect in male mice with a significant reduction in the incidence of mortality (79–27% and 86–20%, resp., for halothane and heat exposure), while leuprolide caused a significant increase in the MH susceptibility of female mice (18–73% and 24–82%, resp., for halothane and heat stress).

**Figure 2:**
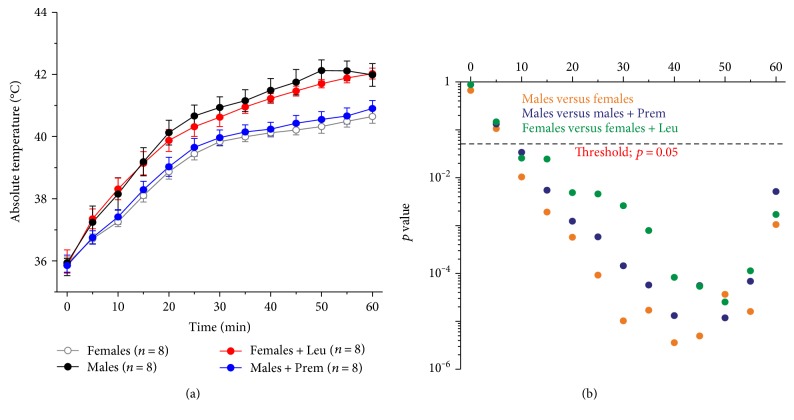
Changes in absolute core temperature in mice subjected to heat stress protocol. (a) Increase in absolute core temperature, recorded every 5 min, during exposure to heat stress protocol in male and female CASQ1-null mice, either untreated or treated with Premarin (males) and leuprolide (females). (b) Semilog plots showing results of repeated measures ANOVA with post hoc Tukey test. Data are given as mean ± SEM; *n* = number of mice. See also Table S3.

As a typical MH crisis is characterized by an abnormal and uncontrolled rise in body temperature, namely hyperthermia [40], we also recorded the rise in core temperature in all mice exposed to the heat stress protocol (Figure 2 and Supplementary Figure 1; see also Supplementary Table 3). Temperature was monitored throughout the entire duration of the experiment, and recorded every 5 min, and showed both as absolute (Figure 2) and relative (ΔT) (Supplementary Figure 1) temperature. The results indicate that, during heat stress protocol, the time-dependent increase in core temperature observed in female mice was significantly lower than that in male mice; specifically, at the end of the stress protocol, the core temperature recorded in females and males was 40.6 ± 0.2°C and 42.0 ± 0.4°C, respectively (Figure 2), with a temperature change from beginning to end of the experiment of ΔT = +4.7 ± 0.3°C and ΔT = +6.1 ± 0.3°C, respectively (Supplementary Figure 1). Following treatment with Premarin, the temperature recorded at the end of the protocol in male mice was 40.9 ± 0.3°C (Figure 2), with an increase of core temperature from beginning to end of the experiment quite similar to that in female mice (ΔT = +5.1 ± 0.2°C) (Supplementary Figure 1); conversely, treatment of female mice with leuprolide resulted in a significantly increased rise in core temperature to values similar to that of male mice, with an absolute temperature at the end of 42.1 ± 0.2°C (Figure 2) and relative increase of +6.4 ± 0.5°C (Supplementary Figure 1).

### 3.2. Estrogens Reduce Muscle Damage and Rhabdomyolysis in EDL Muscles of CASQ1-Null Mice during Heat Stress

**Figure 3:**
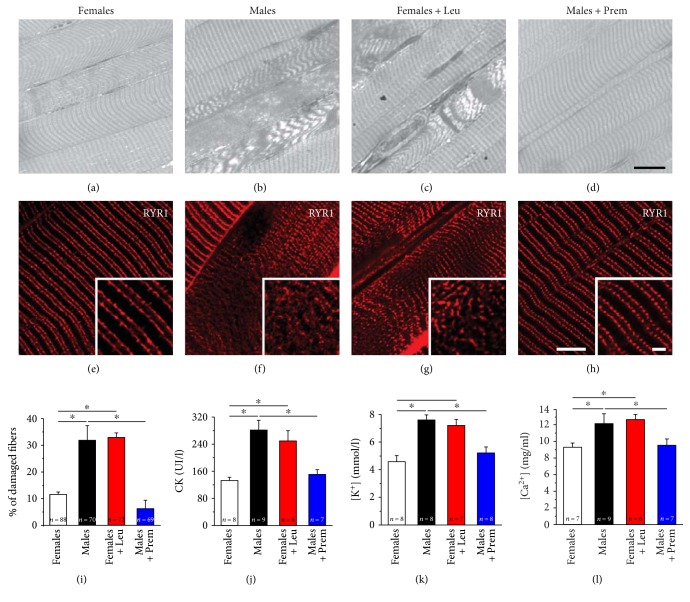
Assessment of muscle damage and blood levels of CK K^+^, and Ca^2+^ following exposure to heat stress protocol. (a–h) Histological (a–d) and immunofluorescence of EDL fibers labeled with anti-RYR1 antibody (e–h) examination of EDL muscles after exposure to the heat stress protocol in male and female CASQ1-null mice, either untreated or treated with Premarin (males) and leuprolide (females). (i) Quantitative analysis of EDL fibers presenting structural damage and contractures. See also Table S4. (j–l) Blood levels of CK in serum (j), K^+^, and Ca^2+^ in plasma (k and l) following heat stress protocol. Data are given as mean ± SEM; ^∗^*p* < 0.05; *n* = number of mice. Scale bars in (a–e): 10 *μ*m (insets 5 *μ*m).

Rhabdomyolysis, a typical clinical sign of MH episodes and exertional/heat strokes in humans [41, 42], is characterized by breakdown of skeletal muscle fibers with the release of the intracellular proteins and ions into the blood stream. We (a) analyzed histological sections to quantify the percentage of EDL fibers affected by structural damage following the heat stress protocol (Figures 3(a), 3(b), 3(c), 3(d), and 3(i); see also Supplementary Table 4); (b) labeled small bundles of EDL fibers with a primary antibody against RYR1, marking the position of calcium release units (CRUs) to visualize striation abnormalities (Figures 3(e), 3(f), 3(g), and 3(h)); (c) measured the blood levels of CK (in serum), K^+^ (in plasma), and Ca^2+^ (in plasma), recognized markers of skeletal muscle damage and rhabdomyolysis (Figures 3(j), 3(k), and 3(l)). Following the heat stress protocol, while normal cross striation was well preserved in fibers from females (Figures 3(a) and 3(e)), fibers from males showed severe disarray of the internal organization, with large areas presenting loss of striation and hypercontracted myofibrils (Figures 3(b) and 3(f)). The effects of treatments were striking: pretreatment of male mice with Premarin strongly protected muscle fibers from heat stress-induced damage (Figures 3(d) and 3(h)), whereas treatment of female mice with leuprolide resulted in a clear increase in the number of fiber presenting loss of striation and contractures (Figures 3(c) and 3(g)).

In histological sections (Figures 3(a), 3(b), 3(c), and 3(d)), we also quantified the percentage of fibers presenting structural damage (Figure 3(i); see also Supplementary Table 4) following the heat stress challenge: 11.6 ± 5.4% of fibers presented signs of structural damage in female mice, while in males, this percentage was 31.9 ± 5.4%. Again, the effect of treatments was striking as EDL fibers from male mice treated with Premarin were protected from structural damage (6.3 ± 3.2%), while the percentage of altered fibers in EDL muscles dissected from leuprolide-treated female mice was increased (32.9 ± 1.7%).

In support of the structural evidence collected by analysis of histological sections (Figures 3(a), 3(b), 3(c), and 3(d)) and by confocal microscopy images (Figures 3(e), 3(f), 3(g), and 3(h)), biochemical analysis of blood samples revealed that the serum and plasma levels of markers of rhabdomyolysis (i.e., CK, K^+^, and Ca^2+^) were lower in female and Premarin-treated male mice (Figures 3(j), 3(k), and 3(l)), but higher in the other two groups of animals.

### 3.3. Estrogens Lower the Temperature and Caffeine Sensitivity of EDL Muscles Isolated from CASQ1-Null Mice

**Figure 4:**
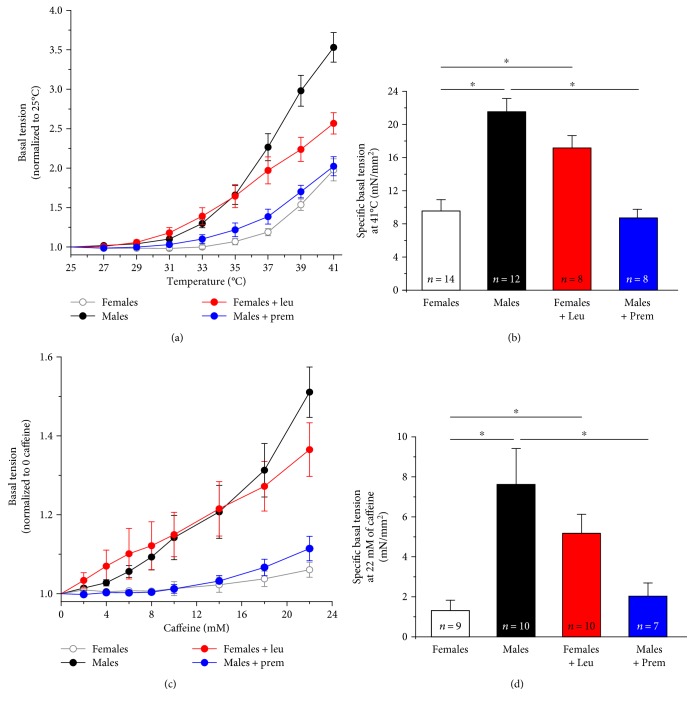
Temperature and caffeine dependence of basal tension in isolated EDL muscles. (a) Temperature dependence of basal tension in EDL muscles excised from male and female CASQ1-null mice, either untreated or treated with Premarin (males) and leuprolide (females). (b) Specific basal tension (expressed as mN/mm^2^) calculated at the last experimental point (T = 41°C). (c) IVCT performed using increasing caffeine concentrations in EDL muscles. (d) Specific basal tension (expressed as mN/mm^2^) calculated at the last experimental point (22 mM caffeine). Data are given as means ± SEM; ^∗^*p* < 0.05; *n* = number of muscles.

To evaluate the effect of increasing temperature on muscle contractility, we performed an *in vitro* heat stress protocol, based on exposure of isolated EDL muscles to increase steps of temperature of 2°C each. When exposed to this protocol, EDL muscles from female mice showed a slight increase in basal tension starting at ~33°C, with a more significant increase in tension only at temperatures above 37°C (Figure 4(a)). On the other hand, EDL muscles excised from male mice started to develop tension already at ~31°C, with the development of strong contractures toward the end of the protocol (Figure 4(a)). Premarin and leuprolide treatments, completely reverted this temperature sensitivity: specifically, Premarin reduced the rise in basal tension in male EDL muscles (Figure 4(a)), with a decrease of specific force calculated at the last experimental point (41°C) of ~40% (Figure 4(b)). Conversely, leuprolide treatment increased the temperature sensitivity of EDL muscles from female mice (Figure 4(a)) with a specific force at the end of the experiment (41°C), ~30% higher than that of EDLs from females (Figure 4(b)).

We then performed a classic caffeine-dose response experiment, mimicking the *in vitro contracture test* (IVCT) that is used in humans to test MH susceptibility [43, 44]. Caffeine is a potent agonist of RYR1 that triggers release of Ca^2+^ from the SR: MH susceptible patients usually display a lower threshold of response to caffeine [45, 46]. The contractile response during IVCT (Figure 4(c)) indicated that EDL muscles from female mice displayed a caffeine sensitivity significantly lower than that from male mice, as clearly shown by the development of tension at lower caffeine concentrations in the latter (Figure 4(c)). Also in this case, Premarin and leuprolide treatments completely reverted the sensitivity of EDL muscles. Specifically, Premarin treatment of male mice (a) increased the threshold of response to higher caffeine concentrations; (b) reduced specific basal tension at 22 mM caffeine of ~73% compared to that recorded in control males; and (c) abolished the development of full contractures (Figures 4(c) and 4(d)) of EDL muscles. On the other hand, leuprolide treatment of female mice (a) lowered the threshold of response to caffeine, with tension that started to rise already at 2 mM caffeine; (b) increased the specific basal tension at 22 mM caffeine of ~75% compared to control females; and (c) caused development of full contractures (Figures 4(c) and 4(d)) of EDL muscles.

In the same muscles, we also evaluated twitch tension in response to increasing caffeine concentrations (Supplementary Figure 2). During the first steps of caffeine application (from 2 to 8 mM), while muscles from females showed an enhancement of twitch force, those from males displayed already a decay, likely due to a faster SR depletion [12]. Again, Premarin and leuprolide treatments completely inverted the ability of EDL muscles to produce force in dependence of caffeine (Supplementary Figure 2). Indeed, muscles from Premarin-treated males exhibited an increased capability to produce force, along the entire range of caffeine application, compared to those of control males, while muscles from leuprolide-treated females displayed a significant caffeine-dependent force decline, very similar to that of males.

### 3.4. Estrogens Normalize Electrical-Evoked Ca^2+^ Transients and Reduce the Caffeine-Induced Ca^2+^ Release in Single FDB Muscle Fibers

**Figure 5:**
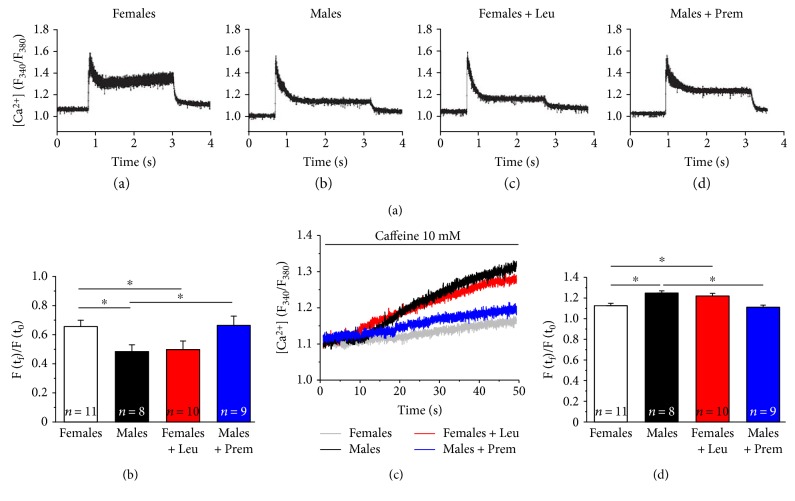
Cytosolic Ca^2+^ concentration during stimulation at 60 Hz and caffeine-induced Ca^2+^ release in single FDB fibers. (a–d) Representative traces of Fura-2 fluorescence obtained during sustained high-frequency electrical stimulation (60 Hz, 2 s) in single FDB fibers from male and female CASQ1-null mice, either untreated or treated with Premarin (males) and leuprolide (females). (e) Fractional Fura-2 ratio signal, calculated as the ratio between the fluorescence peaks at the end (*F* (*t_f_*)) and at the beginning (*F* (*t*_0_)) of the 60 Hz stimulation protocol. (f) Representative traces of Fura-2 fluorescence showing the increase of myoplasmic Ca^2+^ induced by 10 mM caffeine. (g) Fractional Fura-2 ratio signal calculated as the ratio between the fluorescence peaks at the end (*F* (*t_f_*)) and at the beginning (*F* (*t*_0_)) of the protocol. Data in (e) and (g) are given as means ± SEM; ^∗^*p* < 0.05; *n* = number of fibers.

We have previously shown that single FDB fibers from male CASQ1-null mice undergo severe SR depletion when stimulated at high frequency [12]. Here, we measured myoplasmic Ca^2+^ during prolonged high-frequency stimulation (60 Hz, 2 s) and during low-frequency stimulation (0.5 Hz, 0.2 s) in the presence of 10 mM caffeine, in enzymatically dissociated single FDB fibers loaded with the ratiometric Ca^2+^ dye Fura-2. When exposed to a 60 Hz stimulus train for 2 seconds, FDB fibers from female mice displayed a myoplasmic Ca^2+^ transient with a lower decay compared to that observed in male fibers (Figures 5(a) and 5(b)). Specifically, the average residual Fura-2 fluorescence at the end of the 2 sec stimulus (calculated as the ratio between Fura-2 ratio at the end of the stimulation with that recorded at the beginning of the stimulation) was, respectively, 0.62 ± 0.02 and 0.44 ± 0.02 in FDB fibers from female and male mice (Figure 5(e)). Interestingly, in FDB fibers from leuprolide-treated female mice (Figure 5(c)), the Ca^2+^ transient decay was significantly increased compared to that of females (but similar to that of males), with an average value of residual fluorescence of 0.45 ± 0.03 (Figure 5(e)). On the other hand, in FDB fibers from Premarin-treated male mice (Figure 5(d)), the Ca^2+^ transient decay was markedly reduced compared to that of males, with an average value of residual fluorescence of 0.63 ± 0.03 (Figure 5(e)).

As excessive basal tension and development of full contractures are both indicative of abnormally elevated Ca^2+^ levels, we also assessed the caffeine-induced SR Ca^2+^ release in single FDB fibers that were continuously stimulated at low frequency (0.5 Hz) (Figures 5(f) and 5(g)). Consistent with the results obtained during IVCT experiments (Figures 4(c) and 4(d)), FDB fibers from female mice showed a lower caffeine-dependent rise of myoplasmic Ca^2+^ concentration than that observed in FDB fibers from male mice (Figure 5(g)). As expected, while Premarin treatment in males strongly reduced the caffeine-induced SR Ca^2+^ release in FDB fibers, leuprolide treatment resulted in an enhanced elevation of myoplasmic Ca^2+^ concentration in female fibers (Figure 5(g)).

### 3.5. Estrogens Reduce Oxidative Stress in Muscles from CASQ1-Null Mice by Modulating Expression Levels of Either ROS/RNS-Generating or Antioxidant Enzymes

**Figure 6:**
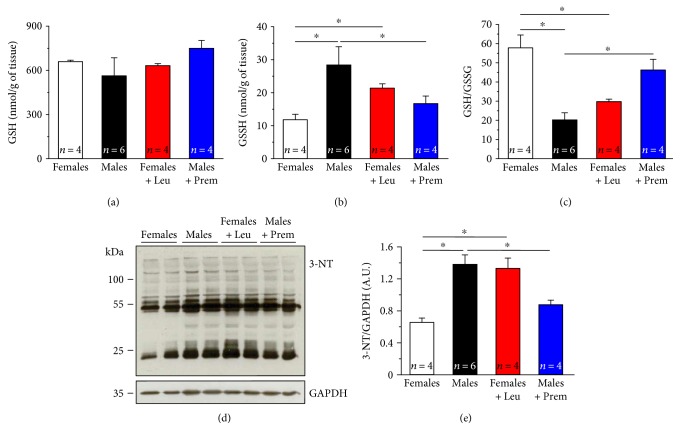
Levels of GSH, GSSG, and 3-nitrotyrosine (3-NT) in both total hind limb and EDL muscle homogenates. (a and b) Levels of reduced (GSH) and oxidized (GSSG) glutathione (both expressed as nmol/g of tissue), measured in control conditions in hind limb muscles from male and female CASQ1-null mice, either untreated or treated with Premarin (males) and leuprolide (females). (c) GSH/GSSG ratio measured in the same specimens. (d) Representative immunoblots showing levels of 3-NT measured in control conditions in EDL muscles from male and female CASQ1-null mice, either untreated or treated with Premarin (males) and leuprolide (females). (e) Relative band densities normalized to GAPDH in the same specimens. Data are given as mean ± SEM; ^∗^*p* < 0.05; *n* = number of mice.

As excessive production of ROS and RNS has been proposed to be a key step in the cascade of molecular events that leads to rhabdomyolysis of muscle fibers and consequent death of MH susceptible mice [15, 28, 29], here, we measured markers of oxidative stress in EDL muscle homogenates. First, we assessed levels of GSH and GSSG (Figures 6(a) and 6(b)), a molecule synthesized from amino acids that is capable of reducing disulfide bonds to cysteines by serving as an electron donor [47, 48], and the GSH/GSSG ratio (Figure 6(c)), a parameter often used as a measure of cellular ROS reactivity [49]. GSH did not differ significantly in the four different groups of mice (Figure 6(a)), whereas GSSG levels were significantly higher in male and leuprolide-treated female mice than those in the other two groups (Figure 6(b)): as a consequence, the GSH/GSSG ratio in female mice was about 3-fold lower compared to male mice, suggesting that in females the global oxidative stress is markedly lower than that in males (Figure 6(c)). Noticeable, after 1 month of treatment, muscles from Premarin-treated male mice exhibited an increase of GSH/GSSG ratio compared to that of males (more than doubled), while muscle homogenates of leuprolide-treated female mice showed a decrease of about 2-fold than those of female mice (Figure 6(c)).

We also measured by Western blot, again in EDL homogenates (Figures 6(d) and 6(e)), the amount of 3-nitrotyrosine (3-NT) (Figures 6(d) and 6(e)), a product of nitration of tyrosine residues of proteins mediated by RNS such as peroxynitrite anion and nitrogen dioxide, which is an indicator of oxidative stress and oxidative protein damage [50]. Whereas in females, 3-NT levels were significantly lower than those in males; after 1 month of Premarin treatment, male mice displayed a significant decrease of 3-NT levels compared to control males (~30%). Conversely, 1 month of leuprolide treatment of female mice determined an increase of 3-NT content of ~30% compared to control females.

**Figure 7:**
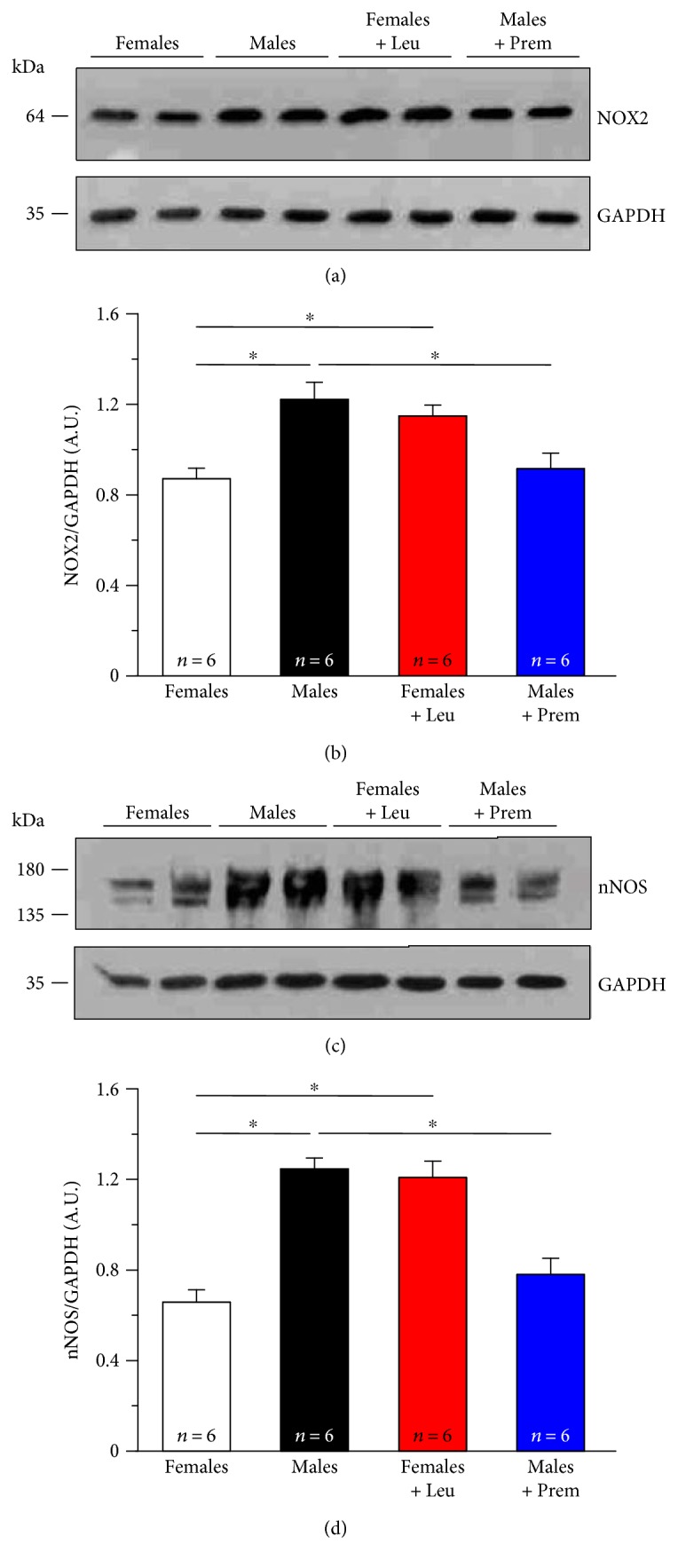
Levels of NOX2 and nNOS in EDL muscle homogenates. (a and c) Representative immunoblots showing levels of NOX2 gp91-phox subunit (a) and nNOS (c) in control conditions in EDL muscle homogenates from male and female CASQ1-null mice, either untreated or treated with Premarin (males) and leuprolide (females). (b and d) Relative band densities normalized to GAPDH. Data are given as mean ± SEM; ^∗^*p* < 0.05; *n* = number of mice.

To further dissect the molecular mechanisms by which estrogens modulate oxidative stress in muscle fibers from CASQ1-null mice, we then evaluated by Western blot the ability of estrogens to regulate expression of either ROS/RNS-generating enzymes or antioxidant enzymes. First, we measured (i) levels of NADPH oxidase gp91^phox^ membrane-bound subunit (NOX2) (Figures 7(a) and 7(b)), belonging to a multiprotein enzyme complex which uses NADPH as a substrate to convert O_2_ to superoxide anion (O_2_^−^) and hydrogen peroxide (H_2_O_2_) and which represents an important extramitochondrial source of ROS in skeletal muscle fibers [51–53] and (ii) levels of neuronal nitric oxide synthase (nNOS) (Figures 7(c) and 7(d)), one of the three isozymes responsible for the production of nitric oxide (NO) that is highly expressed in skeletal muscle [54, 55]. These analyses revealed that NOX2 and nNOS, respectively, responsible for the generation of ROS and RNS, were ~1.5- and 2.0-fold significantly higher in males compared to females (Figure 7), in line with the results showing that oxidative stress is lower in the latter (Figure 6).

**Figure 8:**
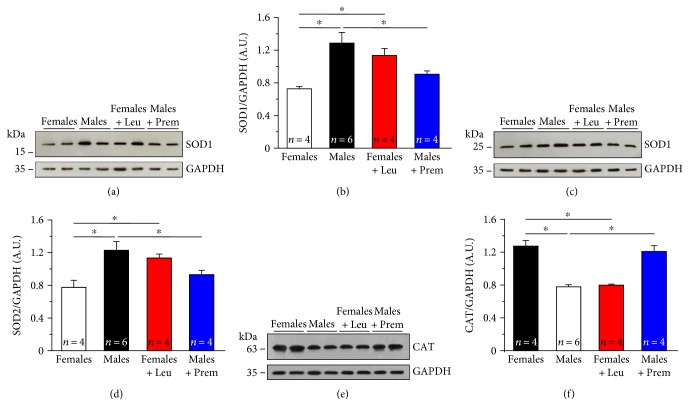
Levels of SOD1, SOD2, and CAT in EDL muscle homogenates. (a, c, and e) Representative immunoblots showing levels of SOD1 (a), SOD2 (c), and CAT (e) measured in control conditions in EDL muscle homogenates from male and female CASQ1-null mice, either untreated or treated with Premarin (males) and leuprolide (females). (b, d, and f) Relative band densities normalized to GAPDH. Data are given as mean ± SEM; ^∗^*p* < 0.05; *n* = number of mice.

Secondly, we evaluated expression levels of (i) copper/zinc superoxide dismutase (SOD1) (Figures 8(a) and 8(b)) and manganese superoxide dismutase (SOD2) (Figures 8(c) and 8(d)), the two main intracellular isoforms of a class of enzymes that catalyze the dismutation of O_2_^−^ into O_2_ and H_2_O_2_, the first step in the elimination of ROS [56] and of (ii) catalase (CAT) (Figures 8(e) and 8(f)), one of the most important antioxidant enzymes that catalyze the decomposition of H_2_O_2_ to H_2_O and O_2_ and that represent an important antioxidant defense for skeletal muscle [57]. We found that while SOD1 and SOD2 were significantly less expressed in female than in male muscles (Figures 8(a) and 8(b); Figures 8(c) and 8(d)), a completely opposite result was obtained for CAT, which was ~1.6-fold more expressed in females than males (Figures 8(e) and 8(f)). Also in these cases, Premarin and leuprolide treatments showed a potent effect in inverting the expression levels in the two genders, of both ROS/RNS-generating enzymes and antioxidant enzymes (Figures 7 and 8).

## 4. Discussion

### 4.1. Background

A review on the epidemiology of MH cases showed a male-to-female ratio of 2.2 : 1 for MHS in humans with males exhibiting a far greater fatality rate [25]. Later studies also found a similar disproportionate male susceptibility, with males representing 78% of the 181 MH cases in the North American MH Registry (NAMHR) [26] and 73% of the 308 NAMHR patients included in a recent MH recrudescence study [58]. Finally, a similar male prevalence (∼4 : 1) was observed in 383 MH cases in Japan from 1961 to 2004 [59].

In 2009, we published a study showing that male mice lacking CASQ1 trigger lethal-hyperthermic episodes with a higher rate of mortality than females when exposed to both halothane and heat, a phenotype resembling human MHS [15]. Here, we hypothesized that female sex hormones may play a role in protecting CASQ1-null animals from MH-lethal episodes and treated male mice with Premarin, a mixture of equine water-soluble estrogens [60, 61], and female mice with leuprolide acetate, a GnRH analog that chronically abolish the hypothalamic-pituitary-gonadal axis [62].

### 4.2. Main Findings

Consistent with the previous work [15], when exposed to halothane and heat stress protocol, CASQ1-null male mice exhibited a higher rate of mortality than female mice, that is, ~4 : 1 ratio. Though, following treatment with Premarin and leuprolide, mortality rate was effectively reverted in the two genders: during exposure to halothane and heat stress, mortality in Premarin-treated males was greatly reduced, while mortality in leuprolide-treated females was significantly increased. The reduced mortality rate in Premarin-treated CASQ1-null male mice was strictly correlated to (a) reduced rise in core temperature; (b) protection from fiber damage; (c) reduced responsiveness of EDL muscles to both temperature and caffeine; and finally (d) reduced SR depletion and increased caffeine-induced Ca^2+^ release. Conversely, the increased mortality rate in leuprolide-treated CASQ1-null female mice was correlated to increased rise in core temperature and fiber damage, enhanced responsiveness of EDL muscles to both temperature and caffeine, and imbalanced Ca^2+^ handling. Our data also showed that EDL muscles from female and Premarin-treated male mice displayed increased GSH/GSSG ratio and reduced levels of nitrated proteins (3-NT) compared to the other two groups of mice, suggesting that estrogens affect global levels of oxidative stress.

### 4.3. Estrogens and Oxidative Stress Levels in Muscle

Durham et al. [28] demonstrated that excessive Ca^2+^-dependent production of ROS and RNS plays a pivotal step in the cellular and molecular events leading to rhabdomyolysis of muscle fibers during MH crises, in RYR1^Y522S/WT^ mice. We also recently reported that treatment of CASQ1-null male mice with antioxidants (i.e., N-acetylcysteine and Trolox) markedly reduced the rate of heat- and halothane-induced mortalities [29]. Finally, the data presented here points to a strict correlation between estrogens, reduced oxidative stress, and protection from MH episodes, as mice with lower levels of oxidative stress also display a reduced mortality, lowered hyperthermia, and protection from fiber damage. The fact that estrogens have potent antioxidant properties is documented in the literature [30, 63], the molecular bases being genomic and nongenomic mechanisms involving their binding to nuclear receptors ER*α* or ER*β* [32] and novel G-protein coupled receptors GPR30 and ER-X localized in the plasma membrane [64, 65]. Also documented is the fact that estrogens may have direct free-radical scavenging properties, because of the similarity in structure to vitamin E [35]. Also, a recent publication showed the protective role conferred by estrogens in the right ventricle function by showing improved contractile [66]. Specifically, reserve in animals with pulmonary hypertension associated with benefits of mitochondrial bioenergetics (these authors demonstrated that estrogens improve the right ventricle contractility by improving mitochondria structure and function and preventing excessive mitochondrial-induced ROS generation).

Although several data reported in the literature demonstrated potent antioxidant properties of estrogens [30–35], the precise mechanisms by which they could modulate oxidative stress in CASQ1-null mice remain unclear.

Our data show that estrogens possibly exert their antioxidant activity by regulating the expression of NOX2 and nNOS, responsible for the generation of ROS and RNS in muscle fibers. Less straight forward is the interpretation of data regarding SOD1 and SOD2, significantly less expressed in muscles from female and Premarin-treated male mice compared to the other two groups of animals, a result though opposite to that of CAT. Data about SOD1 and SOD2 are in agreement with (a) our previous work showing that male CASQ1-null mice exhibited higher levels of SOD1 than the normal [29] and (b) the literature demonstrating that SOD1 and/or SOD2 expression and activity are increased under conditions of high oxidative stress [67–70].

One possible interpretation of these findings could be that in the presence of estrogens, the reduced expressions of both NOX2 and nNOS (and possibly the consequent reduction in generation of O_2_^−^ and NO) would prevent the upregulation of SOD1 and SOD2 expressions and the consequence accumulation of H_2_O_2_. This, together with the concomitant increase of CAT levels, could result in reduction of global oxidative stress (Figure 6).

### 4.4. Ca^2+^ Handling, Oxidative Stress, and Hormones: The Complicated Puzzle Leading to MH Crises in Male CASQ1-Null Mice

Muscle fibers from male CASQ1-null mice display an excessive leak of Ca^2+^ from the SR in basal conditions (i.e., already without exposure to environmental triggers) [15], possibly resulting from RYR1 hyperactivity due to lack of CASQ1 inhibition on the RYR1 open state [9, 10]. Although SR Ca^2+^ leak is clearly the starting event in MH reactions, also other important mechanisms seem to play a pivotal role during the cascade of events leading to rhabdomyolysis of skeletal fibers. Indeed, we have extensively discussed above the involvement of oxidative stress in MH reaction in both RYR1^Y552S/WT^ and CASQ1-null mice [28, 29, 39]. In this puzzle involving imbalanced Ca^2+^ handling and excessive oxidative stress (which are likely not independent from one another), female sex hormones come into play by modulating both parameters. Indeed, in the current study, we showed that estrogens normalize SR Ca^2+^ release by reducing temperature and caffeine sensitivity of EDL muscles during IVCT experiments and decay of electrically evoked SR Ca^2+^ release and caffeine-induced SR Ca^2+^ release. Although the molecular mechanisms by which estrogens normalize intracellular Ca^2+^ handling are still unclear, based on the present and previous studies in MH susceptible mice [15, 28, 29], we are probably entitled to speculate that this could be the consequence of their ability to lower oxidative stress, possibly through the modulation of enzymes like NOX2 and nNOS, involved in the maintenance of redox balance within muscle fibers. Interestingly, it has been demonstrated that (i) both NOX2 and nNOS colocalize with RYRs at the triad junctions [52, 71, 72]; (ii) ROS, generated by NOX2 in the proximity of triads, stimulates SR Ca^2+^ release through RYR1 [52, 73]; and (iii) in cardiac muscle, nNOS is activated by increases in myoplasmic Ca^2+^ concentration, likely due to its colocalization with RYR2 [71, 72], although in skeletal muscle most nNOS localizes in the sarcolemma [74].

Thus, it is possible that, in CASQ1-null muscle fibers, the close positioning of either NOX2 or nNOS to RYR1 and the Ca^2+^-dependent activation of nNOS could be responsible for the excessive production of ROS and RNS which in turn would lead to glutathionylation and nitrosylation of specific cysteine residues [52, 55], oxidative modifications that further increase the opening probability of the leaky RYR1 channel; the consequent excessive release of Ca^2+^ from the SR would promote the dangerous feed-forward mechanism already proposed to underlie MH reactions [28].

### 4.5. Closing Remarks

Although the molecular pathways that allow estrogens to protect skeletal fibers from rhabdomyolysis during MH crises deserve further investigation, the present work contains convincing evidence that female sex hormones do provide an effective protection for CASQ1-null mice against lethal MH-like events. Whether similar mechanisms may underlie also differences in MH incidence between males and females in humans could be worth of consideration.

## Supplementary Material

Supplemental Table 1. Number of mice exposed to halothane (2% for 1 h) and relative experimental outcomes (i.e. survived, sudden death, or delayed death) in female and male CASQ1-null mice, untreated and treated with Leuprolide (females) or Premarin (males). ^∗^*p* < 0.05, compared to sex-matched untreated mice. See also Figure 1. Supplemental Table 2. Number of mice exposed to heat stress protocol and relative experimental outcomes (i.e. survived, sudden death, or delayed death) in female and male CASQ1-null mice, either untreated and treated with Leuprolide (females) or Premarin (males). ^∗^*p* < 0.05, compared to sex-matched untreated mice. See also Figure 1. Supplemental Table 3. Changes in absolute and relative (*Δ*T) core temperature during heat stress protocol, measured at the beginning (t_0_) and end (t_60_) of the experiments, in female and male CASQ1-null mice, either untreated and treated with Leuprolide (females) or Premarin (males). Data are given as mean ± SEM; ^∗^*p* < 0.05, compared to sex-matched untreated mice. See also Figure 2. Supplemental Table 4. Percentage of EDL muscle fibers presenting structural damage in female and male CASQ1-null mice, either untreated or treated with Leuprolide (females) and Premarin (males); ^∗^*p* < 0.05, compared to sex-matched untreated mice. See also Figure 3 I. Supplemental Figure 1. Changes in relative core temperature in mice subjected to heat stress protocol. A) Increase in relative core temperature (*Δ*T), recorded every 5 minutes, during exposure to heat stress protocol (41°C for 1 hr) in male and female CASQ1-null mice, either untreated or treated with Premarin (males) and Leuprolide (females). B) Semilog plots showing results of repeated measures ANOVA with post-hoc Tuckey test. Data are given as mean ± SEM; n = number of mice. See also Table S3. Supplemental Figure 2. Caffeine dependence of twitch tension in isolated EDL muscles. A) Average twitch tension during electrical stimulation (0.2 s at 0.2 Hz applied every 5 seconds; duty cycle: 0.04) at increasing caffeine concentrations. B) Specific twitch tension (mN/mm^2^) at the end of the experiment (22 mM caffeine). Data in A and B have been generated from the same EDL muscles used in Figure 4. Data are given as means ± SEM; ^∗^*p* < 0.05; n = number of muscles.

## Abbreviations

CASQ1: Calsequestrin type-1
CASQ1-null: CASQ1 knockout mice
CAT: Catalase
EC coupling: Excitation-contraction coupling
EDL: Extensor digitorum longus
FDB: Flexor digitorum brevis
GSH: Reduced glutathione
GSSG: Oxidized glutathione
H_2_O_2:_: Hydrogen peroxide
IVCT: *In vitro* contracture test
NO: Nitric oxide
nNOS: Neuronal nitric oxide synthase
NOX2: NADPH oxidase gp91^phox^ membrane-bound subunit
MH: Malignant hyperthermia
O_2_^−^: Superoxide anion
ROS: Reactive species of oxygen
RNS: Reactive species of nitrogen
RYR: Ryanodine receptor
SOD1: Copper/zinc superoxide dismutase
SOD2: Manganese superoxide dismutase
SR: Sarcoplasmic reticulum.
