# Supplementary material for: Estrogens Protect Calsequestrin-1 Knockout Mice from Lethal Hyperthermic Episodes by Reducing Oxidative Stress in Muscle
Source: Oxid Med Cell Longev. 2017 Sep 10;2017:6936897. doi: 10.1155/2017/6936897 (PMC5610815; doi:10.1155/2017/6936897)
Supplement: Supplementary file 3 [file 6936897.f3.pdf]

SUPPLEMENTARY MATERIALS

Halothane sensitivity test

	Females	Males	Females + Leu	Males + Prem
Mice tested	17	14	11	11
survived	14	3	3	8
sudden deaths	3	11	8	3
delayed deaths	0	0	0	0
**% Survived**	**82 %**	**21 %**	***27 %**	***73 %**

Supplemental Table 1. Number of mice exposed to halothane (2% for 1 h) and relative experimental outcomes (i.e. survived, sudden death, or delayed death) in female and male CASQ1-null mice, untreated and treated with Leuprolide (females) or Premarin (males). *$p<0.05$, compared to sex-matched untreated mice. See also Fig. 1.

Heat stress test

	Females	Males	Females + Leu	Males + Prem
Mice tested	17	21	11	20
survived	13	3	2	16
sudden deaths	1	16	8	3
delayed deaths	3	2	1	1
**% Survived**	**76 %**	**14 %**	***18 %**	***80 %**

Supplemental Table 2. Number of mice exposed to heat stress protocol and relative experimental outcomes (i.e. survived, sudden death, or delayed death) in female and male CASQ1-null mice, either untreated and treated with Leuprolide (females) or Premarin (males). *$p<0.05$, compared to sex-matched untreated mice. See also Fig. 1.

Core temperature during heat stress

	Females	Males	Females + Leu	Males + Prem
Mice tested	12	10	8	13
t_0	36.0 ± 0.2	35.8 ± 0.3	35.9 ± 0.2	36.1 ± 0.2
t_{60}	40.6 ± 0.1	42.4 ± 0.2	42.0 ± 0.2	40.9 ± 0.1
**ΔT**	**4.7 ± 0.3**	**6.7 ± 0.3**	***6.2 ± 0.2**	***4.9 ± 0.5**

Supplemental Table 3. Changes in absolute and relative (ΔT) core temperature during heat stress protocol, measured at the beginning (t_0) and end (t_{60}) of the experiments, in female and male CASQ1-null mice, either untreated and treated with Leuprolide (females) or Premarin (males). Data are given as mean ± SEM; *p<0.05, compared to sex-matched untreated mice. See also Fig. 2.

Histological examination of rhabdomyolysis

	Females	Males	Females + Leu	Males + Prem
n° of fibers analyzed	88	72	70	69
damaged fibers	10	23	23	5
**damaged fibers (%)**	**11.4**	**31.9**	***32.9**	***7.2**

Supplemental Table 4. Percentage of EDL muscle fibers presenting structural damage in female and male CASQ1-null mice, either untreated or treated with Leuprolide (females) and Premarin (males); *p<0.05, compared to sex-matched untreated mice. See also Fig. 3 I.

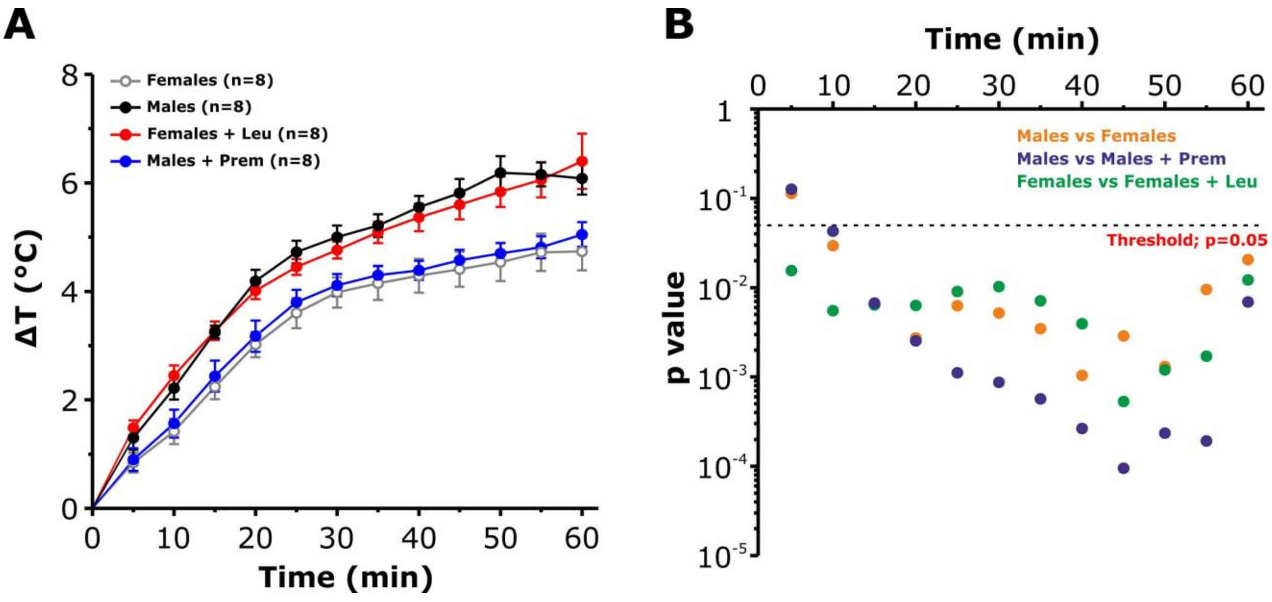

Supplemental Figure 1. Changes in relative core temperature in mice subjected to heat stress protocol. A) Increase in relative core temperature (ΔT), recorded every 5 minutes, during exposure to heat stress protocol (41°C for 1 hr) in male and female CASQ1-null mice, either untreated or treated with Premarin (males) and Leuprolide (females). B) Semilog plots showing results of repeated measures ANOVA with *post-hoc* Tuckey test. Data are given as mean ± SEM; n = number of mice. See also Table S3.

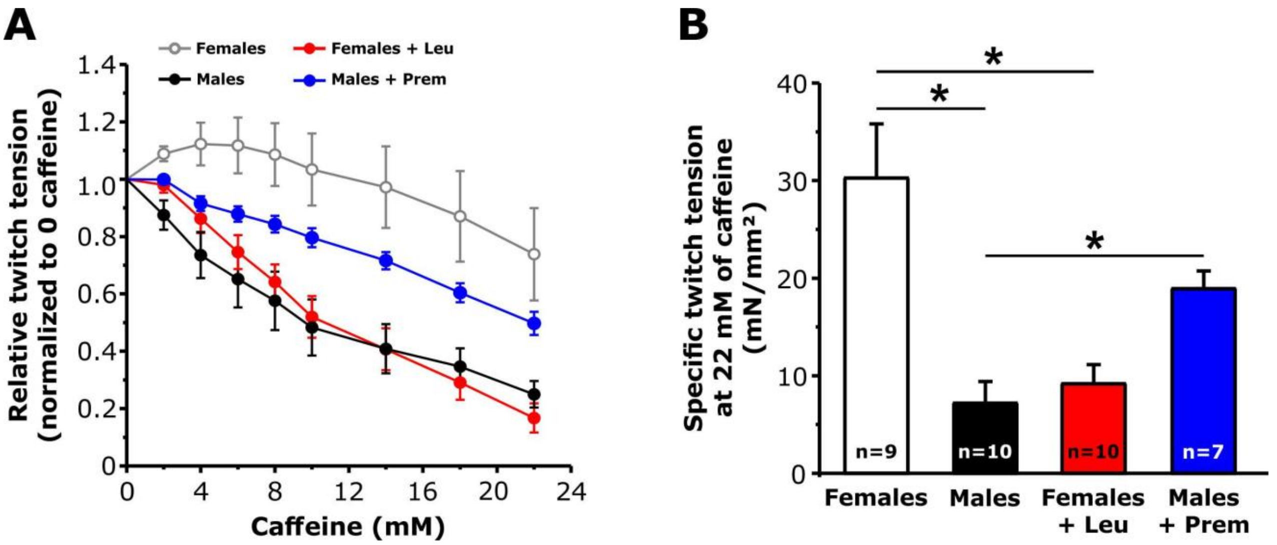

Supplemental Figure 2. Caffeine dependence of twitch tension in isolated EDL muscles. A) Average twitch tension during electrical stimulation (0.2 s at 0.2 Hz applied every 5 seconds; duty cycle: 0.04) at increasing caffeine concentrations. B) Specific twitch tension (mN/mm^2) at the end of the experiment (22 mM caffeine). Data in A and B have been generated from the same EDL muscles used in Fig 4. Data are given as means ± SEM; *p<0.05; n = number of muscles.
