# Supplementary material for: Estrogens Protect Calsequestrin-1 Knockout Mice from Lethal Hyperthermic Episodes by Reducing Oxidative Stress in Muscle
Source: Oxid Med Cell Longev. 2017 Sep 10;2017:6936897. doi: 10.1155/2017/6936897 (PMC5610815; doi:10.1155/2017/6936897)
Supplement: Supplementary file 4 [file 6936897.f4.docx]

**SUPPLEMENTARY MATERIALS**

**Halothane sensitivity test**

|  | **Females** | **Males** | **Females + Leu** | **Males + Prem** |  |
| --- | --- | --- | --- | --- | --- |
| Mice tested | 17 | 14 | 11 | 11 |  |
| survived | 14 | 3 | 3 | 8 |  |
| sudden deaths | 3 | 11 | 8 | 3 |  |
| delayed deaths | 0 | 0 | 0 | 0 |  |
| **% Survived** | **82 %** | **21 %** | ***27 %** | ***73 %** |  |

***Supplemental Table 1.*** Number of mice exposed to halothane (2% for 1 h) and relative experimental outcomes (i.e. survived, sudden death, or delayed death) in female and male CASQ1-null mice, untreated and treated with Leuprolide (females) or Premarin (males). *p<0.05, compared to sex-matched untreated mice. See also Fig. 1.

**Heat stress test**

|  | **Females** | **Males** | **Females + Leu** | **Males + Prem** |  |
| --- | --- | --- | --- | --- | --- |
| Mice tested | 17 | 21 | 11 | 20 |  |
| survived | 13 | 3 | 2 | 16 |  |
| sudden deaths | 1 | 16 | 8 | 3 |  |
| delayed deaths | 3 | 2 | 1 | 1 |  |
| **% Survived** | **76 %** | **14 %** | ***18 %** | ***80 %** |  |

***Supplemental Table 2.*** Number of mice exposed to heat stress protocol and relative experimental outcomes (i.e. survived, sudden death, or delayed death) in female and male CASQ1-null mice, either untreated and treated with Leuprolide (females) or Premarin (males). *p<0.05, compared to sex-matched untreated mice. See also Fig. 1.

**Core temperature during heat stress**

|  | **Females** | **Males** | **Females + Leu** | **Males + Prem** |
| --- | --- | --- | --- | --- |
| Mice tested | 12 | 10 | 8 | 13 |
| t_0_ | 36.0 ± 0.2 | 35.8 ± 0.3 | 35.9 ± 0.2 | 36.1 ± 0.2 |
| t_60_ | 40.6 ± 0.1 | 42.4 ± 0.2 | 42.0 ± 0.2 | 40.9 ± 0.1 |
| **ΔT** | **4.7 ± 0.3** | **6.7 ± 0.3** | ***6.2 ± 0.2** | ***4.9 ± 0.5** |

***Supplemental Table 3.*** Changes in absolute and relative (ΔT) core temperature during heat stress protocol, measured at the beginning (t_0_) and end (t_60_) of the experiments, in female and male CASQ1-null mice, either untreated and treated with Leuprolide (females) or Premarin (males). Data are given as mean ± SEM; *p<0.05, compared to sex-matched untreated mice. See also Fig. 2.

**Histological examination of rhabdomyolysis**

|  | **Females** | | **Males** | **Females + Leu** | **Males + Prem** |  |
| --- | --- | --- | --- | --- | --- | --- |
| n° of fibers analyzed | | 88 | 72 | 70 | 69 | |
| damaged fibers | | 10 | 23 | 23 | 5 | |
| **damaged fibers (%)** | | **11.4** | **31.9** | ***32.9** | ***7.2** | |

***Supplemental Table 4.*** Percentage of EDL muscle fibers presenting structural damage in female and male CASQ1-null mice, either untreated or treated with Leuprolide (females) and Premarin (males); *p<0.05, compared to sex-matched untreated mice. See also Fig. 3 I.


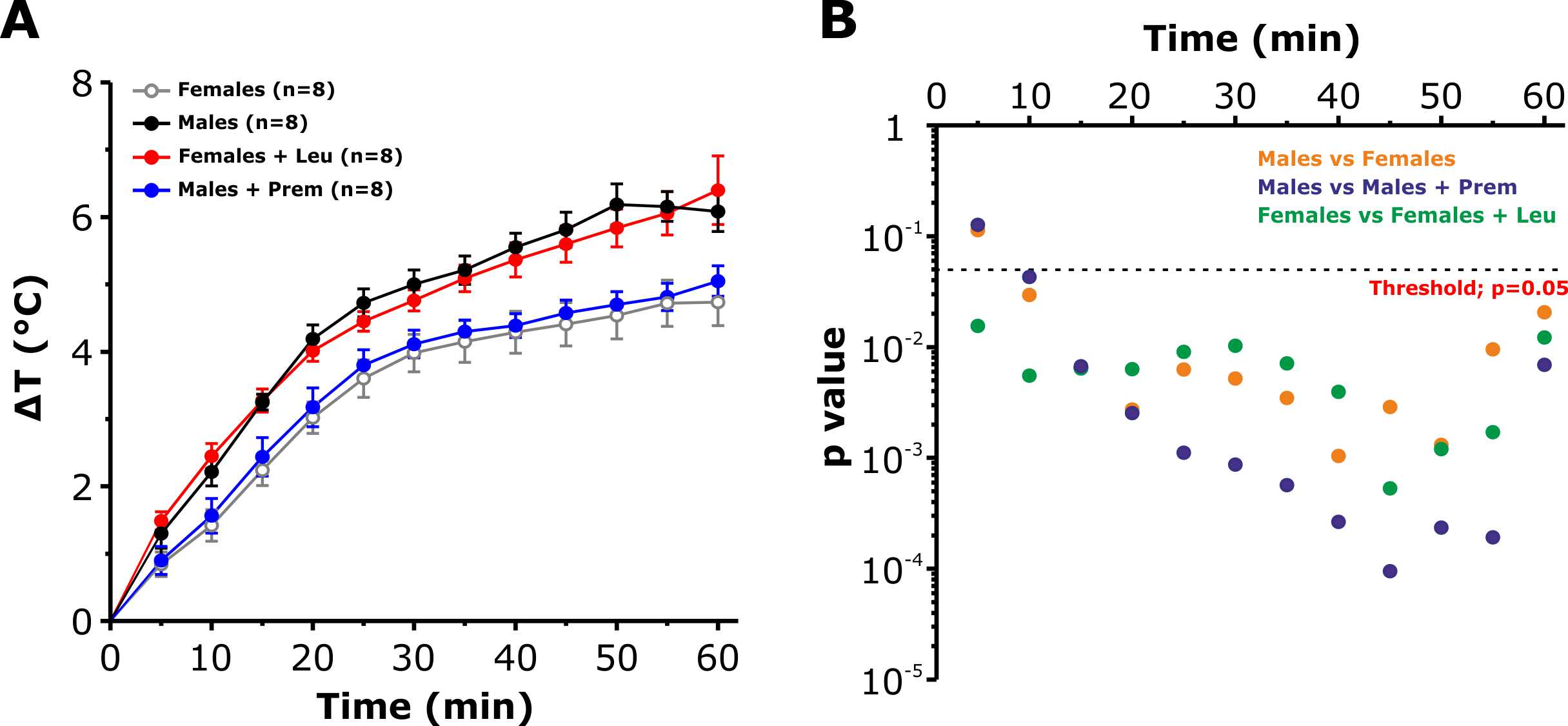


***Supplemental Figure 1. Changes in relative core temperature in mice subjected to heat stress protocol.*** A) Increase in relative core temperature (ΔT), recorded every 5 minutes, during exposure to heat stress protocol (41°C for 1 hr) in male and female CASQ1-null mice, either untreated or treated with Premarin (males) and Leuprolide (females). B) Semilog plots showing results of repeated measures ANOVA with *post-hoc* Tuckey test. Data are given as mean ± SEM; n = number of mice. See also Table S3.


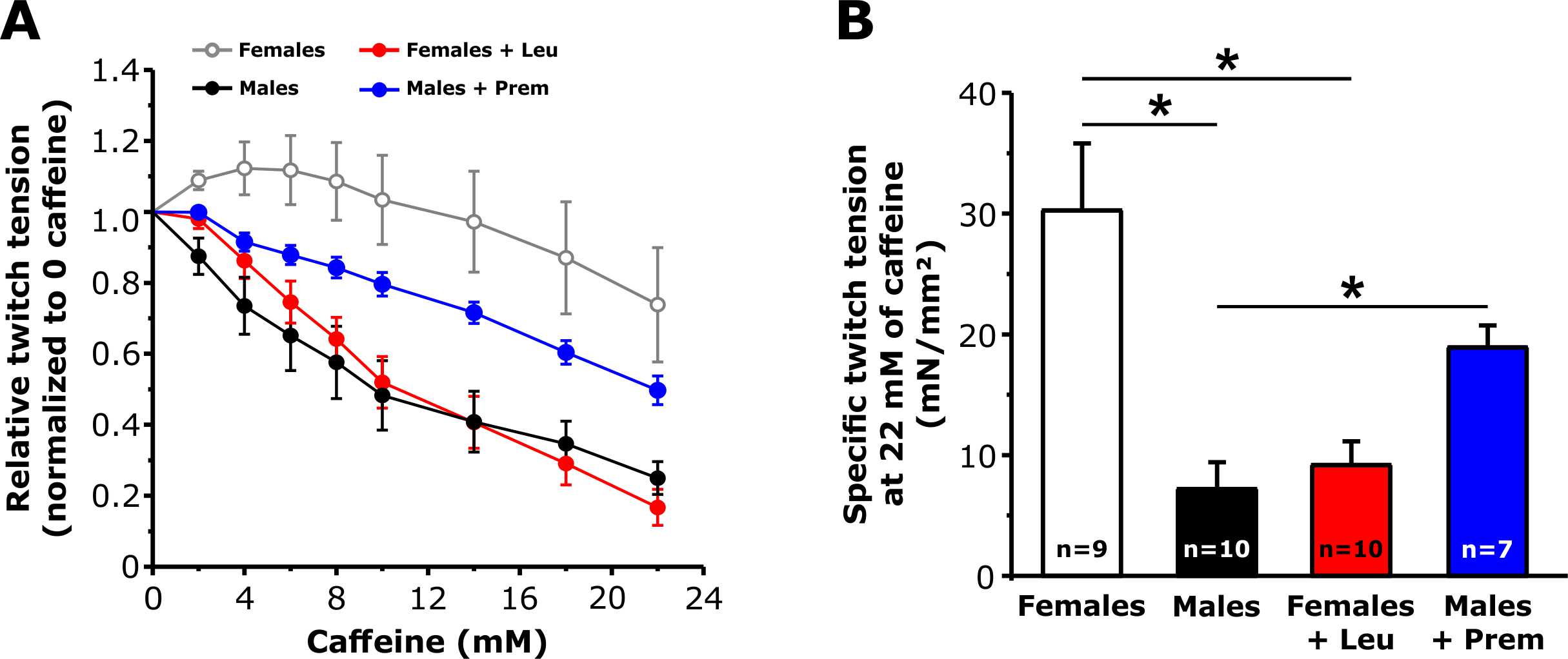


***Supplemental Figure 2. Caffeine dependence of twitch tension in isolated EDL muscles.*** A) Average twitch tension during electrical stimulation (0.2 s at 0.2 Hz applied every 5 seconds; duty cycle: 0.04) at increasing caffeine concentrations. B) Specific twitch tension (mN/mm^2^) at the end of the experiment (22 mM caffeine). Data in A and B have been generated from the same EDL muscles used in Fig 4. Data are given as means ± SEM; *p<0.05; n = number of muscles.
